# Supplementary figures and images for: Isolation and Characterization of Extracellular Vesicles Secreted In Vitro by Porcine Microbiota
Source: Microorganisms. 2020 Jun 30;8(7):983. doi: 10.3390/microorganisms8070983 (PMC7409281; doi:10.3390/microorganisms8070983)

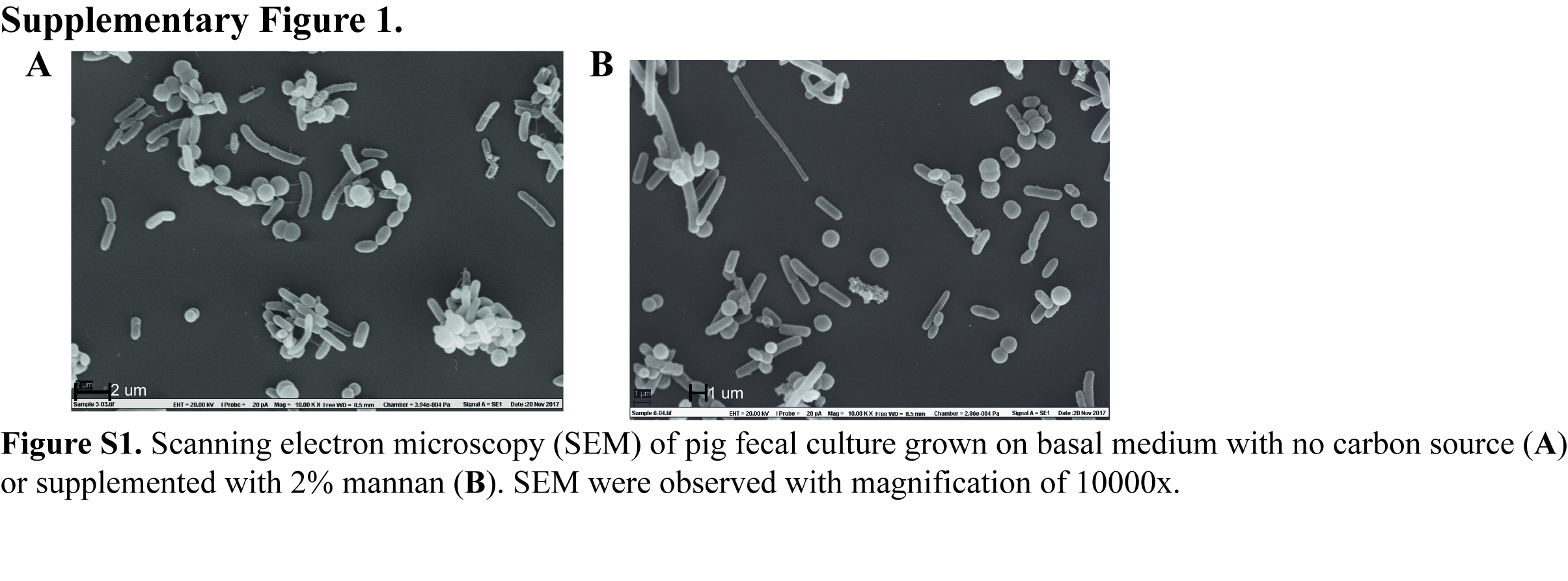

Supplement: Supplementary file 1 [file microorganisms-08-00983-s001.zip › Supplementary Fig.1_25.06.20.tif]

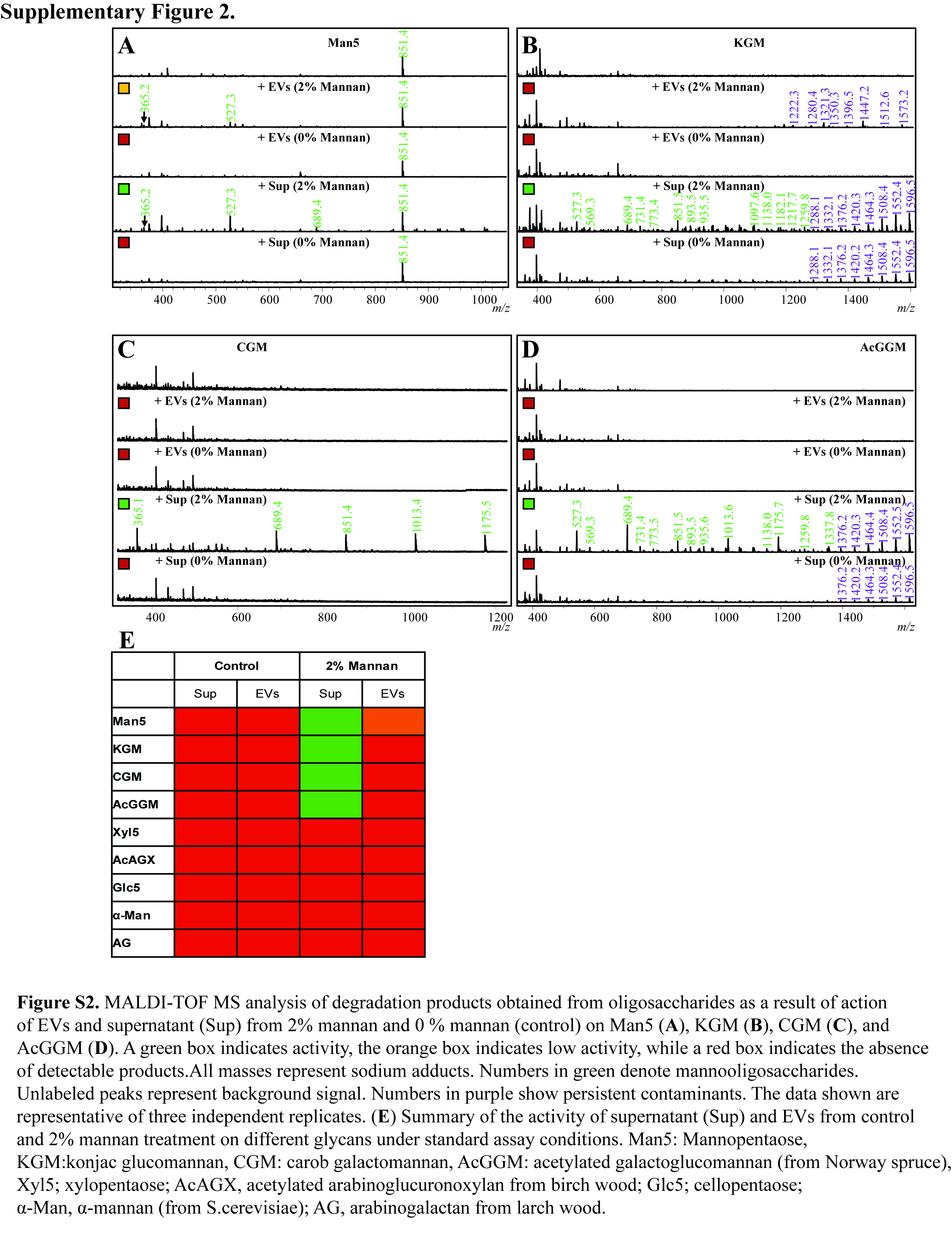

Supplement: Supplementary file 1 [file microorganisms-08-00983-s001.zip › Supplementary Fig.2_25.06.20.tif]
